# Platelet Aggregation Pathway Network-Based Approach for Evaluating Compounds Efficacy

**DOI:** 10.1155/2013/425707

**Published:** 2013-04-04

**Authors:** Jiangyong Gu, Qian Li, Lirong Chen, Youyong Li, Tingjun Hou, Gu Yuan, Xiaojie Xu

**Affiliations:** ^1^Beijing National Laboratory for Molecular Sciences (BNLMS), State Key Laboratory of Rare Earth Materials Chemistry and Applications, College of Chemistry and Molecular Engineering, Peking University, Beijing 100871, China; ^2^Beijing National Laboratory for Molecular Sciences (BNLMS), Center for Molecular Sciences, State Key Laboratory for Structural Chemistry of Unstable and Stable Species, Institute of Chemistry, Chinese Academy of Sciences, Beijing 100190, China; ^3^Institute of Functional Nano, Soft Materials, Soochow University, Soochow Jiangsu 215123, China

## Abstract

Traditional Chinese medicines (TCMs) contain a large quantity of compounds with multiple biological activities. By using multitargets docking and network analysis in the context of pathway network of platelet aggregation, we proposed network efficiency and network flux model to screen molecules which can be used as drugs for antiplatelet aggregation. Compared with traditional single-target screening methods, network efficiency and network flux take into account the influences which compounds exert on the whole pathway network. The activities of antiplatelet aggregation of 19 active ingredients separated from TCM and 14 nonglycoside compounds predicated from network efficiency and network flux model show good agreement with experimental results (correlation coefficient = 0.73 and 0.90, resp.). This model can be used to evaluate the potential bioactive compounds and thus bridges the gap between computation and clinical indicator.

## 1. Introduction

Translational research moves basic biological discoveries from the basic bench into the clinic applications, and it uses the clinic observations to indicate future directions for basic research. The biomarkers which are the molecular, biological, or physical characteristics of a specific physiologic state have an immense impact on the prevention and treatment of diseases. For example, the use of blood pressure and cholesterol as biomarkers for diagnostics and therapies has contributed to a 50 percent decrease in cardiovascular mortality in the USA over the past 30 years [1]. Most biomarkers of a specific physiologic state are related to biological pathways. Therefore, the studies on related pathways could help to find the links between the molecules, pathways, cellular entities, and clinic biomarkers.

Biological pathways are a defined group of biological entities that are organized in a specified order and can perform specified biological functions. Networks based on pathways will play an important role in the development of novel polypharmacological strategies to evaluate compounds which will alter the entire pathway rather than inhibit/activate the single target protein. Since many compounds and enzymes whose biological functions are not explored completely in biological pathways, it is time-consuming and expensive to determine biological functions through biological experiments for each. Therefore, it is highly desired if a computational approach can be developed to address this problem [2, 3].

Along with the progress of system biology, many complex diseases such as cancer, cardiovascular disease, and mental disorders are much more complex than initially anticipated because they are often caused by system-wide multifactors rather than being the result of a single defect [4–8]. System biology provides a platform for integrating multiple components and interactions in health and disease state, while conventional approaches focused on a single event. The biological networks are becoming increasingly important for chemical biology and drug discovery. Analysis of biological networks offers an opportunity for integration of biological complexities and multilevel relationships and provides a new framework to understand the molecular basis of physiological or pathophysiological states. The network-based chemical biology and drug discovery aim to harness this knowledge to investigate and understand the impact of interventions of small molecules in the context of biological networks [9, 10].

Several traditional computational approaches such as pharmacophore, quantitative structure activity relationship, molecular similarity, and molecular docking have been used frequently. However, these methods cannot handle the problems in systems level. In recent years, computational polypharmacology approaches have been developed [11–13]. Encouraged by the successes of using computational approaches to tackle various problems in different biological systems, we have developed a novel and valuable computational approach to evaluate the efficacy of ligands by calculating the influence on pathway network [14].

Platelet aggregation plays an important role in myocardial infarction, thrombosis, stroke, and many other related disorders. Normal platelet aggregation is an essential part of hemostatic process which could protect mammalian from injuries of blood vessels. However, improper hemostatic stimuli in the blood could lead to a series of serious disorders and even death. Therefore, antiplatelet aggregation agents may be useful for regulating the platelet aggregation and treating the relative disorders. In this work, we developed a computational approach based on network efficiency and network flux to evaluate the antiplatelet aggregation activities of active ingredients separated from TCM.

## 2. Materials and Methods

### 2.1. Network Construction and Analysis

**Figure 1:**
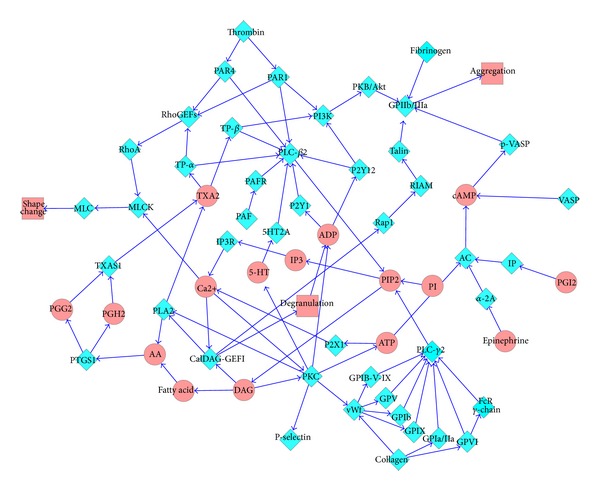
Pathway network of platelet aggregation. Blue diamond and red ellipse represent proteins and small molecules, respectively.

The network was constructed by using the information retrieved from five published literatures [15–19], Reactome [20], and KEGG [21]. The enzymes which participated in the pathway were proposed as nodes, and arrows between nodes represented the connections. The direction of the arrow meant that the node in the end of the arrow was in the downstream of the node in the front. First, Xiang et al. and Broos et al. have constructed the main framework [15, 16]. The information of GPCRs and synthesis of thromboxane was supplemented from other references. Finally, the pathway network of platelet aggregation (Figure 1) contains 64 nodes and 91 edges (arrows).

### 2.2. Multitarget Docking

Nineteen proteins (Table 1) in the pathway network were chosen as targets for docking. The protein-ligand complex structures (crystal or NMR) of each protein were downloaded from RCSB Protein Data Bank (http://www.rcsb.org/pdb/home/home.do). The structures of targets PAR1 and PAR4 were prepared by homology modeling based on crystal structure of bovine rhodopsin (PDB entry: 1U19). The AutoDock4.01 program in DOVIS 2.0 [22] was used for the virtual screening because of the better performance of its scoring function [23]. First, polar hydrogen atoms were added, and nonpolar hydrogen atoms were merged by the hydrogen module in AutoDock Tools (ADTs) for nineteen targets after water molecule was removed. Then, Kollman united atom partial charges were assigned. The grid map of the docking simulation was established by a 40 × 40 × 40 cube centered on the target active site, with a spacing of 0.375 Å between the grid points. When every ligand was docked to a target, the Lamarckian genetic algorithm was used to optimize the conformation of ligands in the binding pocket. The set of parameters was listed as follows: the size of the population was 150. The number of energy evaluations was set to 2.5 × 10^7^ as the run terminates. For clustering the conformations, the root mean square deviation tolerance was 2.0. Twenty independent docking runs were carried out for every ligand. Other parameters were set to default. The original ligands in the complex structures or known inhibitors (Table 1) were used as reference compounds to determine the affinity of compounds to corresponding targets. The compound database used for multitarget docking contained 413 natural products from Chinese herbs which were preserved in our laboratory. Each compound was docked to each target.

### 2.3. Calculation of Network Efficiency and Network Flux

The damage induced by the attacks on the network is characterized by the network efficiency (NE), which is defined as the sum of the reciprocals of the shortest path lengths between all pairs of nodes [24]. Due to a global topological property of a network which could be applied to measure the integrity of the network, the network efficiency was assumed to be used as a measure for drug efficiency [13, 14, 25]. The NE of a graph *G* is measured by the shortest paths between pairs of nodes with the following

(1) $$\begin{array}{c} \text{NE}=\sum_{i\neq j\in G}\frac{1}{d_{ij}}, \end{array}$$

where *d*_*ij*_ is the length of the shortest path between nodes *i* and *j* and the sum is over all *N*(*N* − 1)/2 pairs of nodes with a total number *N* of nodes in the graph *G*. If the network is weighted, *d*_*ij*_ is the path with the minimum weight. The initial edge values of every edge were arbitrarily set to 10. To give a relative network efficiency, this quantity NE is divided by the initial network efficiency. Thus, we considered the network efficiency of the initial network as 100% and measured the relative network efficiency after each attack.

The compounds' effects on the network rely on the docking scores. We supposed that the compounds could inhibit the target well while the docking scores were relatively high. For a ligand, we transformed its docking scores with a target to edge values (EVs) of all direct downstream edges of the target in the network and then calculated the network efficiency. In other words, the edge values of all edges, which point to the other nodes from this target, were reassigned based on the docking score between the compound and the target. The edge values threshold was set to 10, so any edge values which were less than 10 were fixed to 10. We defined that the reference ligand would knock the target by 99.95%. Therefore, the reference ligand could make the value of the edges that come out of the target enzyme as 200. The edge values of the edges which did not come out of the target enzyme were defined as 10. The edge values of the edges in the network were calculated with the follwing

(2) $$\begin{array}{c} \text{EV}=10^{(\text{scor}{\text{e}}_{\text{ligand}}/\text{scor}{\text{e}}_{\text{reference}})\times 2.30}, \end{array}$$

where score_reference_ represents the docking score of the reference ligand, score_ligand_ represents the docking score of other compounds, and EV that are the edge values of the edges, come out of the target in the network. Therefore, different ligands would show different effects on each target. For each ligand, the network efficiency was then calculated using the redefined edge values. The network efficiency of each ligand was ranked by the decrease of the network efficiency. The more the network efficiency decreases, the more potent the ligand would be. The program of network efficiency calculation was written in C++ language using the Dijkstra algorithm.

Network efficiency was a global parameter of a network. However, it could not reflect the different importance of each node in the pathway network. Typically, further down the stream the node is located in a pathway network, the more important it would be. Therefore, we proposed network flux (NF) as a new indicator to evaluate the extent to which compounds influence the pathway network. NF was defined as follows:

(3) $$\begin{array}{c} \text{NF}=\sum_{i\neq j\in G,j\;=\;\text{exit}}\frac{1}{d_{ij}}. \end{array}$$

Network flux was reduced from network efficiency, and NF includes only those shortest paths from upstream node to the exit of the pathway network. The decrease of NE and NF for each compound was listed in Table 2.

### 2.4. Experimental Validation

The inhibition of platelet aggregation induced by ADP was determined by Chrono-log Model 700 Whole Blood/Optical Lumi-Aggregometer (Chrono-Log, Havertown, USA) in Experimental Research Center, China Academy of Chinese Medical Science. First, the blood (4~6 mL per rat) was collected from abdominal aorta, and 10% of (v/v) heparin sodium solution (0.1% dissolved in saline) was added to prevent clotting. Second, the blood was diluted 1-fold with saline for storage. Third, 1 mL of blood was added to the aggregometer and incubated with 10 *μ*L of compound solution (10 mM in DMF) in 37.5°C for 120 seconds. Finally, 10 *μ*L of ADP saline solution (20 *μ*M) was added to the blood, and the inhibition effect was determined by turbidimetric method according to standard protocol. The inhibition effect of each compound was listed in Table 2.

## 3. Results and Discussion

### 3.1. Pathway Network Characteristics

The nodes in the pathway network (Figure 1) covered most of the important enzymes that participated in the process of platelet aggregation, such as GPIIb/IIIa, PAR1, PAR4, PLA2, P2Y1, P2Y12, and PI3K. The network mainly reflected the process of platelet aggregation and was suitable for multitarget virtual screening. Furthermore, the average degree of each node and the average shortest path length were 2.84 and 5.69, respectively. The pathway network showed apparent scale-free property, which meant that this network had strong robustness.

### 3.2. Active Compounds Prediction

The network efficiency could reflect the multitarget interaction of drugs [26]. In order to know the influence by knocking out the 19 docking targets, we measured the network efficiency in fully connected (all edge values were set to 10) or fully blocked (all edge values were set to 99999) state. When there was not any inhibition on the platelet aggregation network, the network efficiency of the whole system was 48.496. To the contrary, the knockout of all the edges that came out from the 19 targets made the efficiency decrease to 8.849.

**Figure 2:**
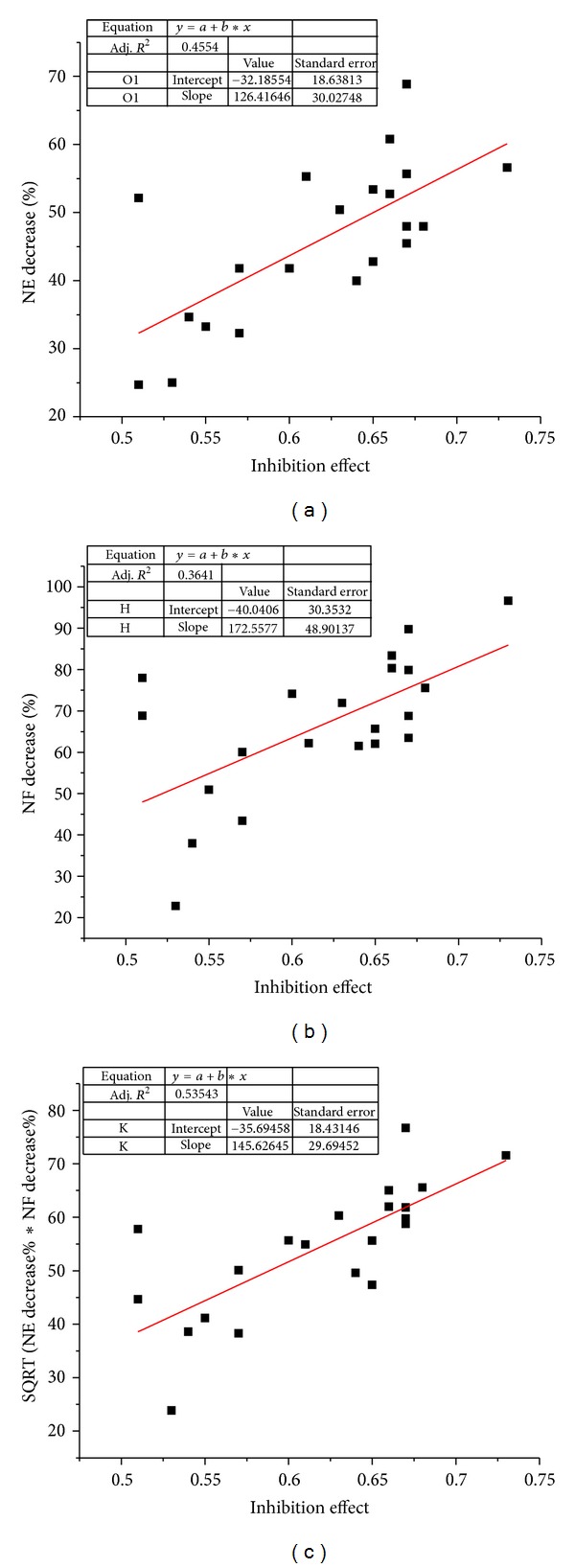
Linear regression between predicated activities of 19 natural products and two drugs and experimental inhibition effects. (a) network efficiency; (b) network flux; (c) combination of network efficiency and network flux.

For each screening compound, we have calculated the network efficiency in which the target proteins were inhibited by this compound. Then, we sorted the 413 screening compounds by the decrease of network efficiency and took out the first 40 compounds for antiplatelet aggregation tests. Experimental results of the whole blood antiplatelet aggregation tests showed that there were 19 compounds with antiplatelet aggregation activities among the 40 compounds, and the percentage of hits was 47.5%. Among the 19 active ingredients, silybin and papaverine were the most two potent molecules that were even comparing favorably with tirofiban which was an effective drug used in treatment of acute myocardial infarction. The papaverine, silybin, and tirofiban could completely inhibit the rat's whole blood platelet aggregation at the concentration of 100 *μ*M. Decrease of the dosage to 34 *μ*M slightly reduced the inhibitory effect, and the inhibitory rates for papaverine, silybin, and tirofiban were 67%, 64%, and 73%, respectively. Furthermore, to examine the quantitative predicting abilities of the model, we also compared the predicted potent value and the experimental results. Numerical analysis showed that the linear correlation coefficient of the decrease of network efficiency and whole blood antiplatelet aggregation experimental results was 0.67 (Figure 2(a)).

The linear correlation coefficient was not very high. This may be because the more important effect the downstream nodes may exert had not been taken into account in the calculation of network efficiency. However, network flux can complement network efficiency. After the addition of NF, the linear correlation coefficient increased to 0.73 (Figure 2(c)).

**Figure 3:**
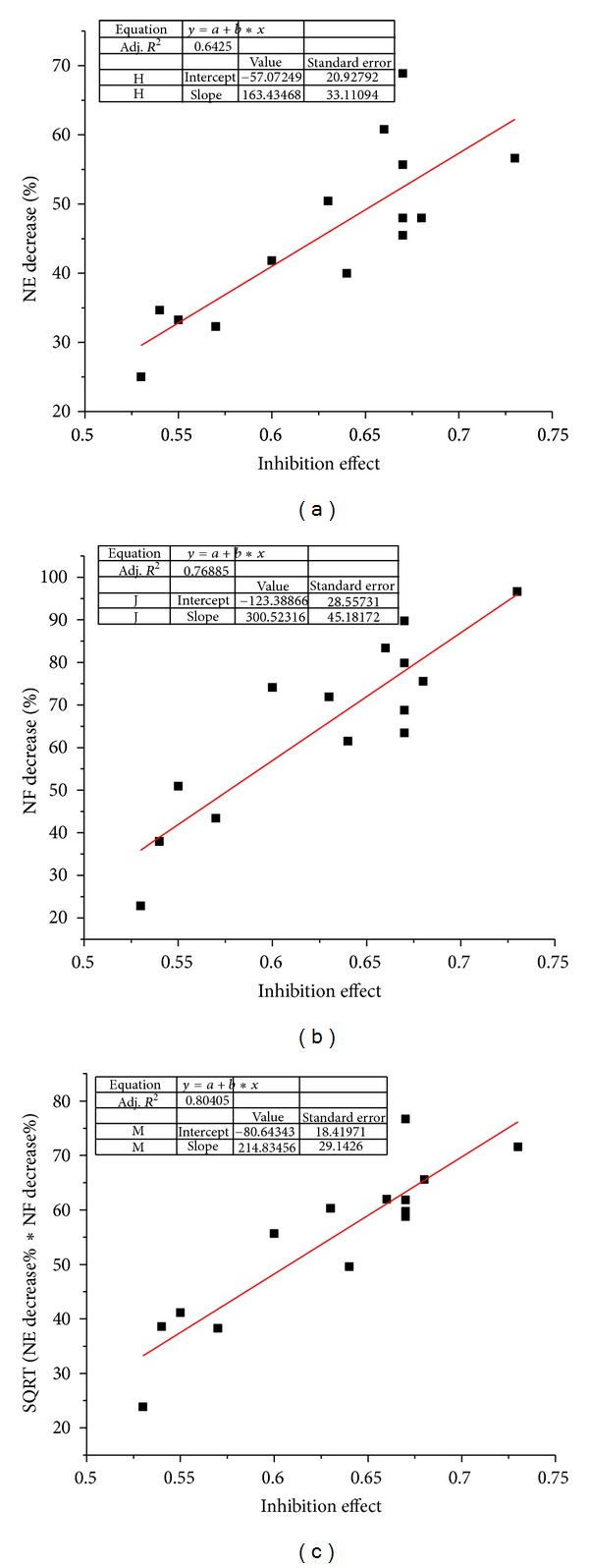
Linear regression between predicated activities of 14 nonglycoside natural products and experimental inhibition effects. (a) Network efficiency; (b) network flux; (c) combination of network efficiency and network flux.

Given that docking program could not treat glycoside with great accuracy because there were many flexible groups, we only considered 14 nonglycoside compounds. In this case, the linear correlation coefficients for the least square fitting of NE, NF, and the combination of NE and NF with experimental results were 0.80, 0.87, and 0.90, respectively (Figure 3). It showed that the combination model could not only qualitatively classify the active and the nonactive compounds but also quantitatively predict the efficacy of the compounds to a certain extent.

Traditional Chinese medicine has been used for thousands of years. TCM presents much diversity in structure and bioactivity, and less toxicity and is an attractive source of new active compounds in drug discovery. The conventional approach to find active compounds in TCM involves selecting a potential plant and isolating compounds following bioassay guidance. This approach has been playing an important part in drug development. However, it was often time-consuming and can contain false positives. We have reported a reverse approach (from finding bioactive molecules to related plant) [27]. Papaverine was isolated mainly from *Papaver somniferum* L. and often used to cause dilation of the blood vessels. Silybin was a flavonolignan isolated from *Silybum marianum* G. with antioxidative and antiinflammatory activities. Therefore, these two herbs would deserve some attention for antiplatelet aggregation activity.

### 3.3. Comparison with Single Target Screening

To reveal the importance of the biological network system in this approach, we compared the predictions generated by single target docking scores with those of network efficiencies and network flux based on the multitarget docking.

The docking results showed that most of the compounds could interact with many targets rather than a single target. When calculated by applying the single target docking scores, the correlation coefficients of the docking score and the experimental data for GPIIb/IIIa, PLA2, P2Y1, and P2Y12, which were approved targets by FDA and supposed as very important targets in platelet aggregation process, were 0.57, 0.20, 0.07, and 0.23, respectively. However, the correlation coefficient between the predictions based on network efficiencies, network flux, and the experimental data was improved by applying the multitarget strategy (*r* = 0.90, Figure 3). It suggested that overall consideration of the contribution of the biological network might be better than only consideration of the contribution of single target for the accurate predictions of the biological activities. The single target docking cannot capture the biological effects of the ligands comprehensively, and the multitarget screening was really necessary to characterize the complicated binding properties of ligands with multiple targets involved in biological network.

Like all virtual screening methods, our approach had many advantages as well as some limitations. One of the obvious advantages of the method was that it specifically considered the role of every target in the whole platelet aggregation process and assigned the weightiness on every target by biological network analysis. The other advantage was that the affinity evaluation in the method was not limited to molecular docking and scoring, as used in this study. Other binding energy prediction methods could also be used, such as pharmacophore, quantitative structure-activity relationship, or comparative molecular field analysis. It was also assumed that the consideration of flexibility of the targets in molecular docking might improve the accuracy of the network efficiency and network flux. On the other hand, one clear disadvantage of this technique was that its accuracy relies heavily on the reliability of network construction and the accuracy of binding affinity assessment.

## 4. Conclusions

In summary, we developed a novel computational approach that combines multitarget docking, network efficiency, and network flux for the predictions of active ingredients separated from TCM with reasonable accuracy. The method integrated the scores generated by the multitarget docking and network analysis in the context of biological pathway. This approach can evaluate the ligands' efficacy more comprehensively than traditional single target docking with much better prediction accuracy and would be very useful for chemical biology and drug discovery. Meanwhile, top two potent compounds (silybin and papaverine) and their source herbs could be promising drugs for antiplatelet aggregation. It remains to be determined what extent and complexity the pathway network takes effect to the biological activity, and the relevant work is underway.

## Figures and Tables

**Table 1:** Nineteen target proteins in pathway network of platelet aggregation.

Protein name	Uniprot	PDB	Ligand^b^	Score^c^
Adenylate cyclase	O60266	1AB8	1AB8	6.38
Glycoprotein IIb/IIIa complex	P05106	2VDM	2VDM	5.29
Inositol 1,4,5-trisphosphate receptor	Q14643	1N4K	1N4K	9.14
P2X purinoceptor 1	P51575	4DW1	4DW1	5.20
P2Y purinoceptor 1	P47900	1Y36	AMP	5.38
P2Y purinoceptor 12	Q9H244	1T78	AMP	3.84
Proteinase-activated receptor 1	P25116	Model^a^	F16357	5.38
Proteinase-activated receptor 4	Q96RI0	Model^a^	YD3	4.28
phosphatidylinositol 3-kinase	P48736	4FUL	4FUL	6.78
RAC-alpha serine/threonine-protein kinase	P31751	3D0E	3D0E	5.39
Protein kinase C	P17252	3IW4	3IW4	6.89
Phospholipase A2	P14555	1J1A	1J1A	5.22
Phosphoinositide phospholipase C beta-2	Q00722	2ZKM	U73122	5.62
Phosphoinositide phospholipase C gamma-2	P16885	2W2W	U73122	5.00
Prostaglandin G/H synthase 1	P23219	3N8X	3N8X	5.05
Ras-related protein	Q9H0U4	3NKV	3NKV	5.30
Thrombin	P00734	3DUX	3DUX	5.02
Thromboxane A2 receptor alpha	P21731	1LBN	SQ29548	7.90
Thromboxane A2 receptor beta	P21731	1LBN	SQ29548	7.90

^a^The structures of targets PAR1 and PAR4 were prepared by homology modeling based on crystal structure of bovine rhodopsin (PDB entry: 1U19).

^b^If the ligand was equal to the PDB entry, the structure was a ligand-protein complex; otherwise the reference ligand was a known inhibitor.

^c^Docking score of reference ligand for each target protein.

**Table 2:** Decrease of network efficiency and network flux, experimental results of each compound.

Compounds	Inhibition^a^	NE decrease %	NF decrease %	Combination of NE and NF^d^
Papaverine	0.67	45.5	79.8	61.8
Tirofiban^b^	0.64	39.9	61.5	49.6
Deoxycholic acid	0.66	60.8	83.4	61.9
Scutellarin^c^	0.51	52.1	77.9	57.8
Rhaponticin^c^	0.65	42.8	65.6	47.3
Dipyridamole^b^	0.60	41.8	74.1	55.6
Chrysin	0.68	47.9	75.5	65.6
Wogonin	0.67	55.6	68.7	58.8
Rhein	0.67	68.8	63.4	59.8
Silybin	0.73	56.6	96.6	71.6
Danshensu	0.57	32.2	43.4	38.3
Quercetin	0.67	47.9	89.7	76.7
Chlorogenic acid	0.54	34.6	37.9	38.6
Icariin^c^	0.65	53.4	62.0	55.6
Quercitrin^c^	0.57	41.8	60.0	50.1
Baicalin^c^	0.61	55.3	62.2	54.9
Liquiritin^c^	0.66	52.7	80.3	65.0
Salvianolic acid C	0.55	33.2	50.9	41.1
Kaempferol	0.63	50.4	71.9	60.3
Salvianolic acid B	0.53	25.0	22.8	23.9
Picroside II^c^	0.51	24.7	68.8	44.6

^a^The inhibition effect determined in the final concentration of tested compounds was 34 μM.

^b^Tirofiban and dipyridamole are two approved drugs.

^c^These seven molecules are glycoside compounds.

^d^Combination of NE decrease and NF decrease: the square root of the product of the percentage of NE decrease and the percentage of NF decrease.
